# Examining the relationship between smartphone characteristics and the prevalence of hand discomfort among university students

**DOI:** 10.1186/s12889-024-20051-5

**Published:** 2024-09-20

**Authors:** Benyamin Rahimian, Faeze Dehghan Banadaki, Fatemeh Moraveji, Sakineh Varmazyar

**Affiliations:** 1 Department of Occupational Health Engineering, Student Research Committee, Faculty of Health, Qazvin University of Medical Sciences, Qazvin, Iran; 2 Department of Occupational Health Engineering, Faculty of Health, Social Determinants Health Research Center and Research Institute for Prevention of Non-Communicable Diseases, Qazvin University of Medical Sciences, Qazvin, Iran

**Keywords:** Pain, Prevalence, Students, Smartphone, Characteristic

## Abstract

**Background:**

Students are among the groups that use smartphones for long periods throughout the day and night. Therefore, this study aimed to examine the relationship between smartphone characteristics and the prevalence of hand discomfort among university students.

**Methods:**

This study included 204 university students, selected based on their willingness to participate and inclusion criteria. Participants reported hand pain and discomfort by completing the Cornell Hand Discomfort Questionnaire (CHDQ). Personal information was collected through a demographic questionnaire. Smartphone characteristics were obtained from the Internet based on the smartphone model self-reported by students.

**Results:**

According to the Cornell questionnaire, 59.3% of students reported experiencing discomfort in their right hand, while 38.2% reported discomfort in their left hand due to smartphone use. Furthermore, 36.3% of students reported experiencing pain in two or more regions on their right hand, while 20.1% reported pain in two or more areas on their left hand. More than half of the students in the right hand (53.5%) and more than one-third (33.3%) in the left hand obtained pain scores of more than 1.5. The chi-square test indicated a statistically significant relationship between the weight of the smartphone and the prevalence of discomfort in the right hand (χ^2^ = 4.80, *p* = 0.03). Furthermore, a statistically significant relationship was found between the discomfort or pain scores experienced in both hands and the number of painful areas in those hands (right hand: χ^2^ = 219.04, *p* = 0.00; left hand: χ^2^ = 213.13, *p* = 0.00).

**Conclusions:**

Smartphone use can cause discomfort and pain in the hands of university students. The physical characteristics of the smartphone, such as its weight, play a significant role in contributing to right-hand-related pain among students. It is important to consider ergonomic factors in smartphone design and usage to reduce musculoskeletal problems among users, especially students.

## Introduction

In today’s world, the rapid advancement of technology has led to an increase in the use of smart devices, especially smartphones [1]. Many students use smartphones for various purposes such as making calls, sending text messages, checking emails, taking photos and videos, chatting on social networks, watching movies, listening to music, searching the Internet, answering calls, and reading university pamphlets [2]. The constant use of smartphones and holding them in their hands can cause college students’ hands to bear the weight of the device.

With the increasing prevalence and duration of smartphone use, the risk of developing musculoskeletal discomfort in various body parts, such as the neck, shoulders, back, and especially the wrists and fingers, is rising. A study conducted at a university in Hong Kong surveyed 503 students using a self-reported questionnaire to assess musculoskeletal consequences related to exposure to electronic devices. The results showed that 251 students (49.9%) reported experiencing discomfort in their upper limbs, shoulders, and neck. Among these students, 155 (61.8%) attributed their discomfort to smartphone usage [1].

In another study conducted in Pakistan among 692 students and university staff, it was found that 438 individuals (63.3%) experienced pain and discomfort in their wrist area [3]. The results of studies indicate that in addition to the duration of smartphone use, the specifications and dimensions of the phone (length, width, thickness, and edge curvature) are significant factors. If these factors do not match and lack ergonomic design, they can lead to musculoskeletal pain in the wrists and fingers [4]. In a study conducted in South Korea, researchers examined the length and width of smartphones in human hands. The results showed that individuals using smartphones with a width of 90 millimeters experienced 12.3% more discomfort and muscle strain compared to those using smartphones with a width of 60 millimeters [5].

In their study, Sahan et al. concluded that younger adults addicted to smartphones reported higher disability scores in the neck region and lower function in their hands [6]. Also, Walankar et al. reported a high prevalence of pain among smartphone users in areas such as the neck, thumb, and lower back. University students reported 44.05% of musculoskeletal pain [7].

Considering the adverse effects of smartphones on physical health [8], especially on the hands that are heavily engaged, it seems essential to raise awareness about the issues related to hand discomfort. Subsequently, providing necessary solutions related to fit, and design of the smartphone can help prevent, reduce, or eliminate these problems.

Studying the associations between smartphone characteristics and the prevalence of hand discomfort helps enhance understanding of factors related to hand discomfort in the university student population. By gaining insights into how smartphone characteristics may contribute to reducing hand issues, this research can inform the development of ergonomic design guidelines and interventions to promote the health and well-being of college students who extensively use smartphones. Therefore, this study aimed to examine the relationship between smartphone characteristics and the prevalence of hand discomfort among university students.

The study aims to identify the specific smartphone specifications that have a greater impact on the prevalence of musculoskeletal pain and discomfort in the hands, fingers, and wrists. Therefore, users should prioritize these specifications when choosing a smartphone. In addition, what changes should smartphone manufacturers implement in the design and production of their devices to improve user-friendliness and compete successfully in the global market?

## Methods

### Samples and the inclusion criteria

The university’s ethics review board approved the study protocol under the code IR.QUMS.REC. 1402.051 and assigned it the contract number 402,000,000. Using the census method, 221 students out of the university’s total student population of approximately 2200 people participated in the study. They were randomly selected based on their willingness to cooperate. Of the 221 college students willing to participate, 17 were found to be ineligible based on the inclusion criteria. Therefore, a total of 204 subjects took part in the study.

The study’s inclusion criteria were as follows: participants had to provide all required information when completing the questionnaire, use a smartphone for at least two hours daily [9], not have any congenital skeletal-muscular abnormalities in the hands [10], no history of hand surgery [9], no history of wrist joint fracture [3], absence of trauma, or injuries to the wrist in the past six months [11], no carpal tunnel syndrome [3], absence of inflammatory arthritis [3, 12], and work for less than 2 h per day on a computer.

Students who completed the consent form were selected to participate in the study. Subsequently, a demographic questionnaire was administered to collect information on age, weight, height, marital status, education level, smartphone model, and inclusion criteria.

### Smartphone characteristics, Cornell Hand Discomfort questionnaires (CHDQ), and statistical analysis

**Fig. 1:**
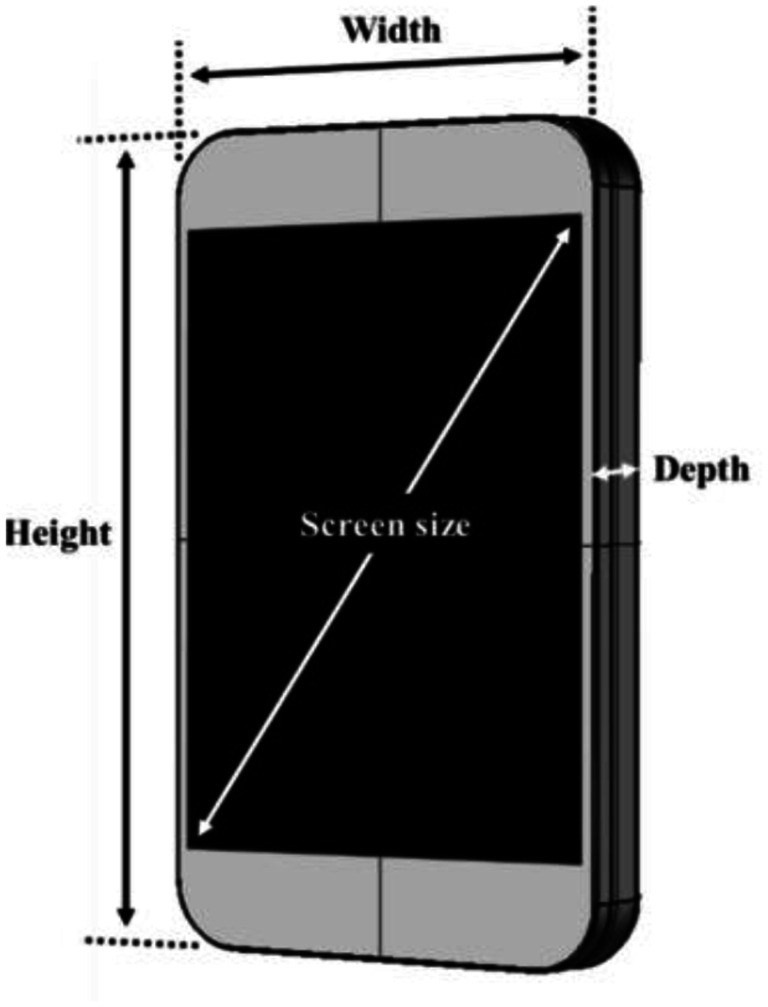
The dimension of smartphones determined in the study based on different models

The smartphone characteristics such as length/height (centimeters), width (centimeters), thickness/depth (millimeters), screen size (centimeters), and weight (grams) were determined from the literature (Fig. 1) [2]. These measurements were based on the smartphone models students recorded in the questionnaire.

**Fig. 2:**
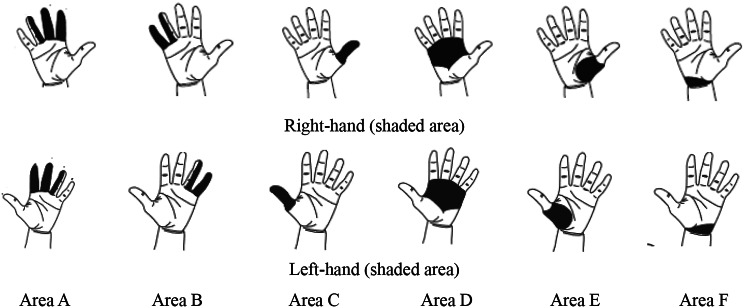
Right-and left-hand map diagram according to the Cornell questionnaire

The Cornell Hand Discomfort Questionnaire (CHDQ) assesses the frequency of discomfort in the fingers, palms, and wrists of both the right and left hands. This validated and reliable questionnaire [13, 14] was obtained with permission from the Human Factors and Ergonomics Laboratory at Cornell University, and has been widely used in previous research [15, 16]. In addition, a Farsi version of this questionnaire for both hands is available on the Cornell University Ergonomics website. The CHDQ divides each hand into six areas for assessment (Fig. 2) [14] and employs a scoring system where the frequency of discomfort is rated on a scale ranging from “never” (score of 0) to “several times every day” (score of 10) [11, 16].

The data analysis used the Chi-square test to examine the relationship between categorized smartphone characteristics and the prevalence of discomfort in each hand (pain-free versus experiencing pain). The cut-off points for smartphone characteristics were determined based on the variables mean. Additionally, the Chi-square test was used to evaluate the association between scores of aches, discomfort, or pain (≤ 1.5, 1.6–14, and ≥ 15) in the hands and the number of reported pain areas (none, one area, two areas, or more) [16].

## Results

The study results showed that 97.1% of the students were single. The participants had a mean age of 21.3 years with a standard deviation of 2.04 years. On average smartphones used by students weigh approximately 186 g. 78.9% of the students were studying at the Bachelor’s level, while 21.1% used both hands while working with their smartphones. Additional information can be found in Table 1.

**Table 1:** Descriptive statistics of quantitative and qualitative information for participating students (*n* = 204) and characteristics of the used smartphones

Quantitative information		Qualitative information			
Variable	Mean ± SD	Variable	Category	Frequency	Percent (%)
Age (years)	21.3 ± 2.04	Marital status	Single	198	97.1
Height (cm)	171.15 ± 9.60		Married	6	2.9
Weight (kg)	67.16 ± 13.77	Gender	Male	82	59.8
Smartphone length (cm)	15.82 ± 0.71		Female	122	40.2
Smartphone width (cm)	7.44 ± 0.53	Education level	Bachelor’s	161	78.9
Smartphone thickness (mm)	8.25 ± 0.59		Master’s	5	2.5
Smartphone screen size (cm)	16.11 ± 1.03		Doctoral	38	18.6
Smartphone weight (gr)	185.95 ± 17.70	Hand-used for smartphone	Right hand	140	68.6
-	-		Left hand	21	10.3
-	-		Both hands	43	21.1

**Fig. 3:**
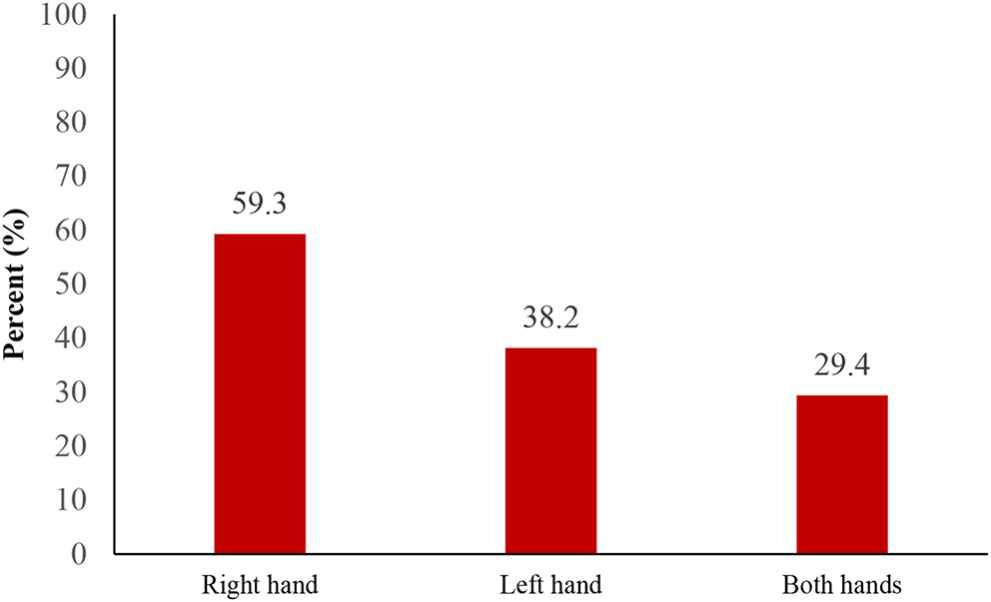
The percentage of pain prevalence in the right hand, left hand and both hands (*n* = 204)

Figure 3 shows that 59.3% of students reported experiencing pain in their right hand, while 38.2% of participants experienced discomfort in their left hand.

**Fig. 4:**
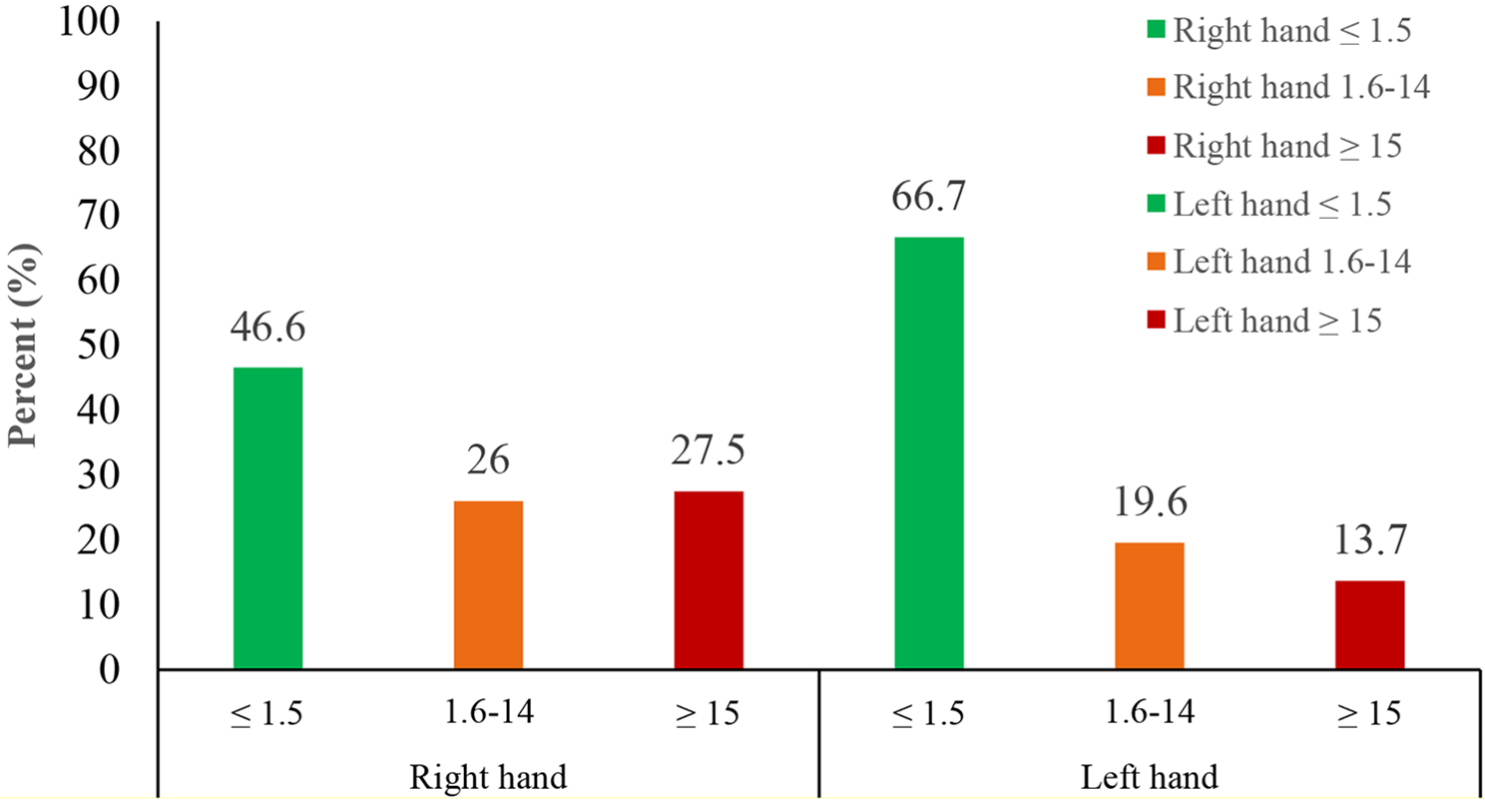
The percentage of the total Cornel questionnaire score classified in the right and left hands (*n* = 204)

The Cornell questionnaire total score results show that 46.6% of participants reported the minimum score (indicating the lowest pain level) in their right hand. In comparison, 66.7% of students reported the minimum score in their left hand (Fig. 4). In other words, the remaining students reported higher scores indicating discomfort in their hands.

**Fig. 5:**
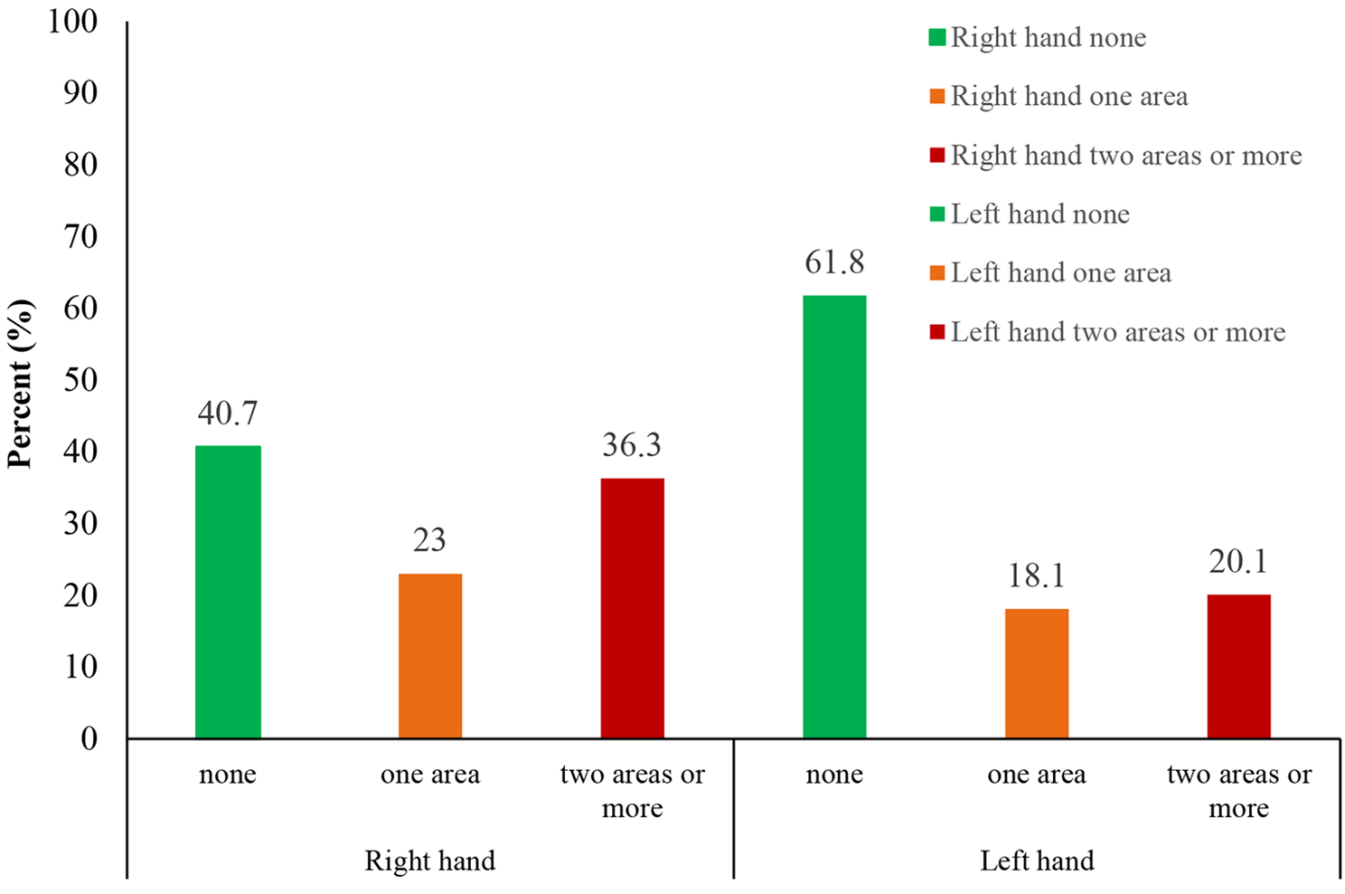
The prevalence of pain based on the number of different pain areas in the right and left hands (*n* = 204)

The results also showed that 36.3% of students experienced pain and discomfort in two or more areas of their right hand, while 20.1% reported pain in two or more areas of their left hand (Fig. 5).

Based on the chi-square test, it was determined that the weight of the smartphone is one of the influential factors causing right-hand pain. Moreover, a statistically significant relationship was discovered between the scores for each hand and the number of reported pain areas (Table 2).

**Table 2:** Results of the investigation into the relationship between smartphone characteristics and the prevalence of hand discomfort, as well as hand discomfort scores and the number of pain areas based on the chi-square test (*n* = 204)

Independent variable Dependent variable	Category based on mean	Percent each category		The prevalence of pain (pain-free or experience pain)									
				Right hand					Left hand				
				χ^2^		df	*p*-value		χ^2^		df		*p*-value
Smartphone length (cm)	≤ 15.82	26.5		0.34		1	0.92		0.44		1		0.59
	> 15.82	73.5											
Smartphone width (cm)	≤ 7.44	32.8		0.70		1	0.15		0.90		1		0.01
	> 7.44	67.2											
Smartphone thickness (mm)	≤ 8.24	56.9		0.36		1	0.85		0.63		1		0.23
	> 8.24	43.1											
Smartphone screen size (cm)	≤ 16.11	29.4		0.45		1	0.57		0.19		1		1.65
	> 16.11	70.6											
Smartphone weight (gr)	≤ 185.95	49.0		0.03*		1	4.80		0.61		1		0.26
	> 185.95	51.0											

Independent variable Dependent variable	Category based on scores	Number of different pain areas (none, one area, two areas or more)											
		Right hand						Left hand					
		χ^2^	df		*p*-value			χ^2^		df		*p*-value	
Discomfort or pain scores in hands	≤ 1.5	219.04	4		0.00**			213.13		4		0.00**	
	1.6–14												
	≥ 15												

*P ≤ 0.05 ** *P* ≤ 0.01

## Discussion

This research examined important information about the relationship between smartphone characteristics and the prevalence of hand discomfort among university students.

Due to smartphone usage, at least one-third (29.4%) of students reported experiencing pain in both hands. Specifically, more than half (59.3%) reported pain in the right hand while more than one-third (38.2%) recorded discomfort in the left hand (Fig. 3). These findings suggest that prolonged smartphone use can result in notable musculoskeletal issues, particularly in the dominant hand used for smartphone interaction. The relatively higher rates of pain reported in the right hand may be due to greater strain or overuse than the non-dominant left hand. This could be a result of more frequent and intensive use of the right hand for activities like typing, contacting, screen movement, searching, etc. Contrary to the current study, Mustafaoglu et al.‘s study results indicated that 68.7% of participants reported wrist/hand pain due to smartphone addiction [11]. The discrepancy in findings may be attributed to variations in the study population and the use of different questionnaires. In addition, the study conducted by Mustafaoglu et al. included participants from a wider age range and implemented various criteria for entry and exit. These factors may have influenced the discrepancies noted in the reported findings. In the study by Amjad et al., 63.3% of students reported experiencing discomfort in their wrist area [3]. Yixin et al. reported a 21.7% frequency of hand pain in the wrist region among smartphone users [17]. The variation in results between the current study and previous studies could be attributed to the different types of smartphones and their specifications.

Other factors that influence the differences between the results of the present study and those of other studies [18, 19] include variations and limitations in the analysis of the duration of daily smartphone use, different postures used, the number of years the smartphone has been used, the number of phones used, the range of work activities carried out by individuals, the number of fingers used while operating the smartphone, the type of smartphone usage, marital status, education level, considering the control group in the study, gender, smoking status, and more. Future studies can explore all these aspects and examine their correlation with the prevalence of hand pain.

The results of the CHDQ showed that over half of the students (53.5%) reported pain scores above 1.5 in their right hand, indicating a moderate to high level of discomfort or pain. Furthermore, one-third of the students (33.3%) reported ache scores above 1.5 in their left hand (Fig. 4). A score above 1.5 indicates that the students experienced discomfort, aches, or pain in their hands 3–4 times in the last week, once daily, or multiple times each day. This highlights the significant impact of smartphone usage on the physical well-being of students. Amjad et al. reported that 22% of individuals experienced wrist pain and disability at a moderate to severe level [3]. The discrepancy in these results could be attributed to the use of different methods and variation in reporting discomfort in the specific area of ​​the hand.

The data also revealed that 36.3% of students experienced pain and discomfort in two or more areas of their right hand, while 20.1% reported aches in two or more regions in their left hand (Fig. 5). This suggests that prolonged smartphone usage can result in more widespread musculoskeletal problems, potentially affecting various hand regions, including the fingers, palm, and wrist.

In this study, no significant association was found between the prevalence of hand pain and smartphone dimensions. Similarly, research conducted by Amjad et al. in 2020 also found that wrist pain was not significantly related to mobile phone screen size [3]. In contrast to the study by Lee et al., which concluded that changing the width of the smartphone from 90 to 60 millimeters resulted in 12.3% of young individuals experiencing more discomfort and muscle strain [5]. In addition, Walankar et al. showed in a logistic regression analysis that the size of a smartphone plays a significant role in predicting musculoskeletal pain [7].

The study found a statistically significant relationship between the weight of a smartphone and the prevalence of discomfort in the right hand. This indicates that the physical characteristics of a smartphone, especially weights of 185.95 g and higher (which account for 51% of university students) may potentially contribute to hand-related problems among smartphone users. In line with the present study, Choi et al.‘s research found that some smartphone models weighed 180 g or more [2]. Based on the results of the current study, it is essential to prioritize ergonomic smartphone design, with a specific focus on weight, to address these issues. In their article, Choi et al. emphasized that the placement of hard keys on smartphones should be ergonomically designed to enhance hand performance [20]. Intolo et al., in their research, recommend smartphone app usage to reduce musculoskeletal pain in different body regions including the hand [21].

## Conclusion

Smartphone usage among college students often leads to hand pain, especially in the hand that is used more frequently typically the right hand. Scores reported on the Cornel questionnaire showed a statistically significant relationship with pain in two or more areas of both hands. Among the smartphone characteristics examined, it was found that weight significantly contributes to discomfort in the right hand. Therefore, users especially students who frequently use smartphones, should be careful when selecting their devices, especially when considering the weight. These findings highlight the importance of addressing ergonomic challenges linked to smartphone use, particularly among university students who heavily rely on these devices for academic and personal activities.

## Limitation and suggestion

One limitation of the current study was the failure to examine the number of finger movements, especially those of the thumb when using smartphones. Since repetitive movements are an important risk factor in the development of musculoskeletal problems, it is recommended that future studies investigate this risk factor and its impact. In addition, future studies could examine the relationship between the dominant hand used by participants and the prevalence of pain more precisely through statistical analysis.

Given the rates of hand pain reported by at least one-third of student participants, there is a clear need for additional research into potential contributing factors. These factors may include prolonged static postures, repetitive movements, and inadequate ergonomic support.

Implementing ergonomic interventions in the future, training on healthy hand and wrist habits, and promoting awareness of musculoskeletal problems may help decrease this issue among the college student population.

## Data Availability

The data in this article will be shared at a reasonable request by the corresponding author.
